# Climate variability rather than overstocking causes recent large scale cover changes of Tibetan pastures

**DOI:** 10.1038/srep24367

**Published:** 2016-04-13

**Authors:** L. W. Lehnert, K. Wesche, K. Trachte, C. Reudenbach, J. Bendix

**Affiliations:** 1 Faculty of Geography, Philipps-University of Marburg, Deutschhausstr. 10, 35037 Marburg, Germany; 2 Senckenberg Museum of Natural History Görlitz, P.O. Box 300154, 02806 Görlitz, Germany; 3 German Centre for Integrative Biodiversity Research (iDiv) Halle-Jena-Leipzig, Deutscher Platz 5e, 04103 Leipzig, Germany

## Abstract

The Tibetan Plateau (TP) is a globally important “water tower” that provides water for nearly 40% of the world’s population. This supply function is claimed to be threatened by pasture degradation on the TP and the associated loss of water regulation functions. However, neither potential large scale degradation changes nor their drivers are known. Here, we analyse trends in a high-resolution dataset of grassland cover to determine the interactions among vegetation dynamics, climate change and human impacts on the TP. The results reveal that vegetation changes have regionally different triggers: While the vegetation cover has increased since the year 2000 in the north-eastern part of the TP due to an increase in precipitation, it has declined in the central and western parts of the TP due to rising air temperature and declining precipitation. Increasing livestock numbers as a result of land use changes exacerbated the negative trends but were not their exclusive driver. Thus, we conclude that climate variability instead of overgrazing has been the primary cause for large scale vegetation cover changes on the TP since the new millennium. Since areas of positive and negative changes are almost equal in extent, pasture degradation is not generally proceeding.

Degradation of grasslands is a global threat for human welfare^1^. In China, estimates of rangeland degradation vary, and as much as 90% of the grasslands is considered to be degraded^2^. Within China, the Tibetan Plateau (TP) forms the world’s largest high mountain grassland ecosystem and is a globally important “water tower”^3^. Depending on the assessment methods that are used to investigate pasture degradation on the TP, the changes have been found to be the result of either climate change or human-induced overgrazing^4^. Conclusive evidence for both effects is, however, lacking^5^, especially regarding their spatial configuration.

Climatic controls are pronounced in the high and often dry region on the TP, where the ecosystems are generally vulnerable to climate change^6^. It was recently shown that vegetation changes alter the sensible heat fluxes and causes feedbacks to the atmosphere, which demonstrates the feedback between climate change and land cover on the TP^7^. Because the TP has been grazed by wild herbivores over evolutionary relevant time scales, the vegetation is generally well-adapted to grazing^8^. However, land use on the TP is now undergoing significant changes because of the sedentarization of the Tibetan nomads, which has had large effects on livestock stocking numbers and pasture use rights. This might exacerbate pasture degradation and the associated loss of water regulation and supply functions and is discussed as key threats to the TP^2^,^4^,^9^. To assess the impact of changes of climate and grazing on pasture condition accurate high-resolution data are needed.

As a consequence of pasture degradation, net biomass and plant cover are reduced^10^, which is demonstrated on the TP by grazing enclosure experiments^11^,^12^ and remote sensing^13^. In the grazing enclosure experiments, a relatively short response time of the ecosystem to grazing was found (*<*10 years)^8^,^12^. The large spatial extent of the TP makes field investigations and the monitoring of plant cover as an indicator for climate and LN changes extremely expensive. Thus, remote sensing approaches provide the only opportunity to investigate recent and widespread changes in plant cover on a spatially explicit basis.

To overcome these spatial deficits and to delineate regions of change since 2000, we use a recently generated MODIS-based high-resolution and wide area dataset of vegetation cover^14^ (see the Methods section). By comparing the trends in air temperature identified from the ERA-Interim reanalysis data^15^, precipitation from the tropical rainfall measuring mission (TRMM, 3B42)^16^ and livestock numbers (e.g., sheep, goats, and yaks; hereafter LN), we identify the drivers of vegetation change. This approach allows us to provide area-wide and region-specific evidence of the interactions and feedbacks between vegetation, land use and climate change on the TP.

## Results

### Changes in livestock numbers on the Tibetan Plateau

**Figure 1:**
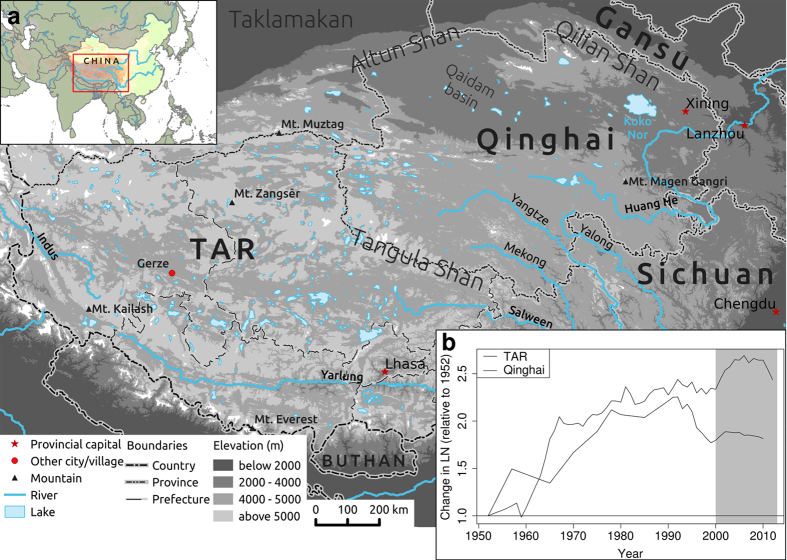
Map of the TP (**a**) and changes in livestock numbers (LN) in the TAR and Qinghai in sheep equivalents (relative to year 1952, **b**). The map shows all of the locations mentioned in the text. The map has been created in R statistical software^40^ based on elevation data, political boundaries, lakes and rivers from^41^. The grey area in (**b**) is the investigated time period during which the satellite data, and thus the area-wide plant cover product, were available.

**Figure 2:**
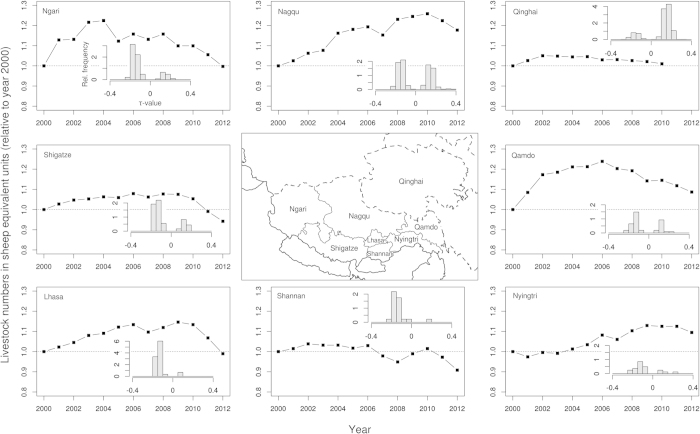
Livestock numbers (LN) in prefectures of the TAR and Qinghai in sheep equivalents (relative to year 2000). The histograms show the relative distributions (in percentages) of significant plant cover trends by prefecture (TAR) or provincial (Qinghai) area. The map has been created in R statistical software^40^ based on political boundaries from^41^.

The major part of the TP belongs administratively to two different provinces, Qinghai in the north-eastern part and the Tibetan Autonomous Region (TAR) covering the southern and western part (see Fig. 1a for a location map). Since regional politics and social structures are considerably different, it is worthwhile to separately analyse LN in both provinces to find out if they evolved differently during the last decades. In the TAR, LN increased dramatically between 2000 and 2006, while LN in the adjacent Qinghai Province decreased considerably during the 1990s and have remained almost constant since 2000 (Fig. 1b). In the TAR, where data for all prefectures are available, LN increased at the beginning of the new millennium in prefectures Ngari and Qamdo, whereas the increase occurred later in Nagqu, Lhasa and Nyingtri (Fig. 2). Only Shigatze and Shannan were not exposed to increasing LN since 2000.

### Changes in precipitation, temperature and vegetation cover between 2000 and 2013

**Figure 3:**
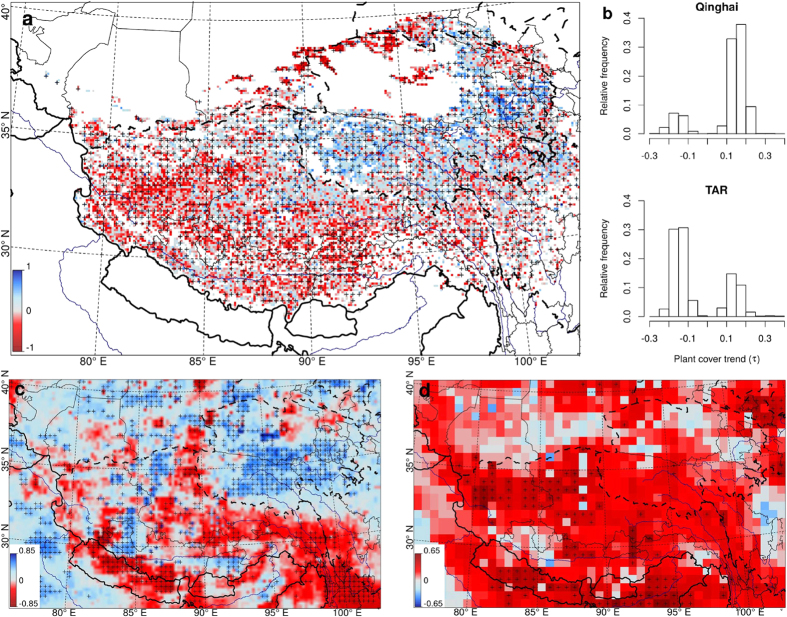
Trends in vegetation cover and climate variables between 2000 and 2013. (**a**) Plant cover trends during the growing season. (**b**) Histograms of the relative frequencies of significant plant cover trends in Qinghai and the TAR (significance level of 0.05). Note that the distributions are bimodal because only significant changes are considered. Maps in (**c**) and (**d**) show trends in precipitation sums and the mean 2 m air temperature, respectively. The colours indicate the *τ*-values of the Mann-Kendall correlations, and the (+) labels mark areas where the correlations are significant at the 0.05 confidence level. For interpretation note that all of the correlations were calculated from the anomalies of the variables. The maps and the histograms have been created using R statistical software^40^.

The vegetation cover increased significantly between 2000 and 2013 along a large belt that encompasses southern Qinghai, the headwater region of the Yangtze and the eastern part of Qinghai (Fig. 3a). The largest increase was observed south of Lake Koko Nor. In contrast, the vegetation cover in the western and southern parts of the TAR has decreased, with the strongest negative trends being observed in the upper reaches of the Indus River. The south-eastern part of the TP does not show clear trends. The comparison of the accumulated vegetation cover trends for grid squares from Qinghai and the TAR highlight the different patterns in each area, with more positive vegetation cover changes for Qinghai and more negative vegetation cover changes for the TAR (Fig. 3b). For the prefectures of the TAR, predominantly negative trends have been observed for Ngari, Shigatze, Lhasa, and Shannan (Histograms in Fig. 2). Qamdo, Nyingtri and Nagqu featured less pronounced patterns.

Precipitation has increased significantly in southern Qinghai and along a small north-south trending band at 85° E (Fig. 3c). Significant negative trends were observed in the upper reaches of the Mekong and Salween rivers in northern Yunnan.

Temperatures increased across nearly the entire TP between 2000 and 2013 (Fig. 3d). However, significant positive trends were only observed in the western part of the TAR, to the south of the Himalayas and north of Qilian Shan. No significant negative temperature trends were found.

**Figure 4:**
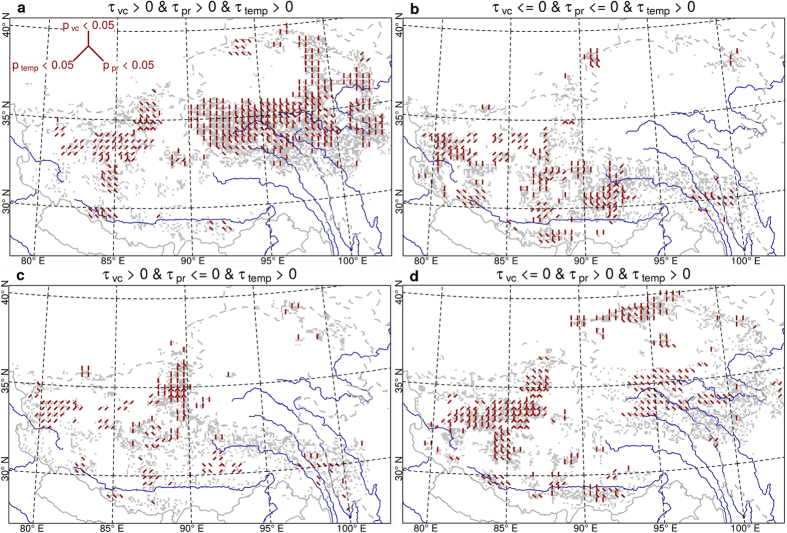
Interactions between vegetation cover and climate variables. (**a**) Positive trends in vegetation cover, precipitation and temperature. (**b**) Positive trends in temperature and negative trends in vegetation cover and precipitation. (**c**) Positive trends in vegetation cover and temperature and negative trends in precipitation. (**d**) Positive trends in precipitation and temperature and negative trends in vegetation cover. The red lines mark areas where the correlations are significant at the 0.05 confidence level (see inset in **a** for an explanation of the lines). The maps have been created using R statistical software^40^.

Positive trends in the climate variables and plant cover were observed in southern Qinghai and the northern part of the TAR (Fig. 4a). Simultaneous occurrences of negative precipitation and plant cover trends were found in the far western part of the TAR and in some areas in the central TAR (Fig. 4b). Positive plant cover trends corresponded to negative precipitation trends at the northern border between Qinghai and the TAR (Fig. 4c) while the co-occurrence of the opposite trends was observed in the western part of the TAR and in the Qilian Shan (Fig. 4d).

## Discussion

The results show unexpected and pronounced differences in the temporal trends of vegetation cover on the TP that are accompanied by differing trends of the potential atmospheric forcings. The trends differ between the provinces of Qinghai and the TAR, which may reflect differences in either land use policy or individual climate elements. Both explanations are theoretically plausible. On the one hand, LN and thus potential overgrazing are strongly influenced by regional land use policies, which differ in the two provinces in that the sedentarization programs began earlier in Qinghai. During these sedentarization programs, the land use system and the storage numbers changed dramatically leading to severe degradation at least in some areas^5^,^17^. On the other hand, the border between the provinces parallels the Tanggula mountain ridge, which may also function as a climatic divide between the southern and northern TP.

**Figure 5:**
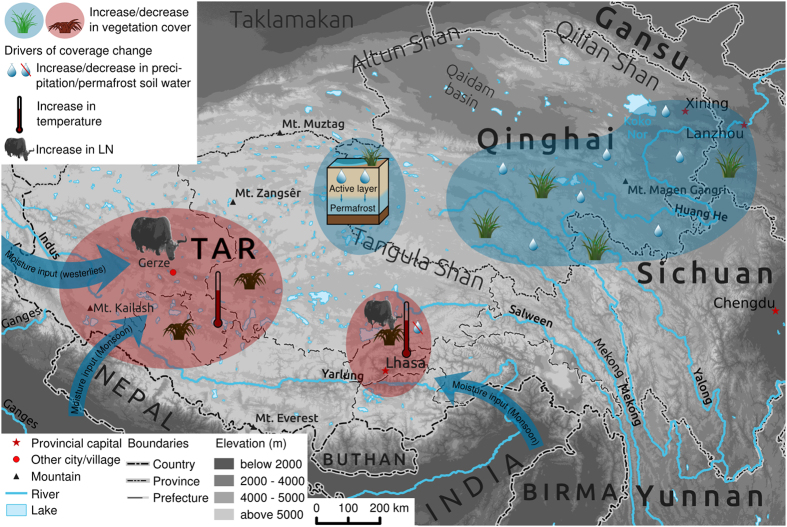
General synopsis of the drivers of significant vegetation cover changes by area. The map shows the conceptual model of land use (anthropogenic degradation) and climatic impacts on vegetation cover (see the text for further discussion). Blue represents areas with dominant positive cover changes, while red indicates negative trends. Blue arrows show the main atmospheric moisture transport paths towards the TP, which are driven by the monsoon system and the extratropical westerlies. The figure has been created using R statistical software^40^. Elevation data, rivers, lakes, and boundaries are from^41^.

Changes in vegetation cover can be caused by changes in climate, such as precipitation, temperature, or a combination of both depending on which is the main local limiting factor for vegetation growth (See Fig. 5 for a summary of the dominant drivers discussed in the following). Precipitation and vegetation cover are positively correlated on a global scale^18^, which has also been confirmed along transects in Inner Mongolia^19^,^20^ and across the TP^21^. Thus, the significant increase of vegetation cover between 2000 and 2013 in southern and eastern Qinghai can be explained by the positive trend in precipitation in these regions. In the TAR, there are only a few areas in which precipitation changes can be the reason for the vegetation trends. Negative precipitation trends correspond to declining vegetation cover in the valleys of Salween and Mekong in eastern Tibet, central Tibet around Lhasa and in the area north of the border with Nepal. The progressive melting of permafrost soils due to local warming could result in an indirect increase of plant available water^22^. This effect might counter-balance the negative precipitation trends and has been demonstrated in the region at the south-western border between Qinghai and the TAR, where the permafrost soils began melting after 2000^23^. This is confirmed by a significant increase in air temperature in the respective region, especially in the summer (Supplementary Table 1), and in the increasing number of months with a mean air temperature above 0 °C (Supplementary Fig. 1).

Significant positive temperature trends have only been observed in the central and north-western TAR, where the vegetation cover is generally declining. Because former permafrost soils have already melted in these arid regions and only discontinuous permafrost islands remained^24^, it is unlikely that thawing permafrost soils provided additional water for the vegetation. In this case, the available water did not change, but the increasing air temperature accelerated evapotranspiration, which may have caused water stress on the vegetation and led to the observed decrease in vegetation cover as recorded in field experiments in central Tibet^25^.

The changes in land use, which are indicated by increasing stocking densities, contribute partly to the predominantly negative trends in the vegetation cover of the TAR. In the western (Ngari prefecture) and to a lesser extent in the eastern (Qamdo prefecture) parts of the TAR, the negative trends in the vegetation cover are the result of the increasing LN as well as the less suitable environment for plant growth. In other areas of the TAR, such as the northern central region (Nagqu prefecture), the effects of increasing LN are apparently mitigated by improving climatic conditions facilitating plant growth. Since the dominant degradation pattern did not differ between prefectures with and without large changes in LN (Histograms in Fig. 2), we conclude that climate variability instead of overgrazing has been the primary driver for degradation changes between 2000 and 2013.

A reduction of plant cover in the TAR is alarming because it leads to higher erosion, lower water retention capacity of the soils, and thus to an acceleration of extreme runoff 26. These direct negative impacts may be further exacerbated because the reduction of vegetation cover significantly feeds back to atmospheric processes^7^,^27^,^28^ by means of a reduction of transpiration and thus latent heat fluxes. This causes an increase in the sensible heat fluxes, which may accelerate convective precipitation processes according to the catalysis hypothesis if moist air masses are advected at higher atmospheric levels; this can occur even if local water recycling is reduced by a loss of vegetation cover^29^, such as due to overstocking. Evidence for this mechanism is provided by the finding that the dominant fractions of atmospheric moisture transport towards the TP occur in the western and south-western parts of the plateau^30^,^31^, where negative vegetation cover and positive precipitation trends were observed. This interrelationship led to an increase in higher intensity convective rainfall, while the average precipitation decreased. Thus, the spatiotemporal patterns of precipitation indicates that the water cycle of the TP has been altered. The observed feedback mechanism may contribute to a higher frequency of extreme precipitation events in southern China and may impact the monsoonal system^32^.

In summary, our study clearly documented recent changes in the grassland vegetation on the TP. While increasing precipitation facilitates plant growth and may counterbalance potential degradation of grassland in large parts of Qinghai, the increase in LN in the TAR, the rising temperatures and the slight decrease in precipitation have resulted in an alarming reduction of grassland plant cover in the western and central parts of the TP. Since the area featuring positive vegetation changes almost equals the area with negative changes, the grassland vegetation on the TP is, however, not subject to a generally ongoing degradation. In contrast to the positive changes, the negative ones occurred dominantly in areas with low productivity. Thus, the overall productivity of the Tibetan pastures has presumably shown a net increase since the year 2000.

Regarding the human influence since the beginning of the new millennium, it has to be clearly stated that LN only caused decreasing vegetation cover if other environmental factors got less suitable for plant growth, as well. However, our data did not allow to assess whether grazing effects had not been more pronounced several decades ago, implying that recent governmental grazing control policies have already had impact. In any case, vegetation cover must be adequately monitored in the future, to ensure that sustainable countermeasures can be taken. This is critically important because an ongoing degradation could have negative effects on the stability of the ecosystem of the Tibetan pastures. This may affect the political stability of the TP region and human prosperity in the densely populated areas of south and south-east Asia that strongly depend on the proper water regulation of the TP.

## Methods

### Vegetation cover

A time series (from 2000 to 2013) of a novel vegetation cover product for grasslands on the TP was used to investigate the recent changes in vegetation dynamics^14^. The new product covers the entire TP and includes plant cover estimates of all grassland vegetation types (See Fig. 1a for a location map). The data are derived from MODIS BRDF composites (MCD43A4) with a spatial resolution of 500 m and a temporal resolution of 16 days. The vegetation cover values are calculated from a cascade of satellite data of increasing spatial resolutions using support vector machine (SVM) regression models, which are trained and validated against plant cover surveys from more than 600 field plots that span the entire TP.

It has been reported that the Terra MODIS sensor is suffering from degradation^33^. This implies that the reflectance values that are used for plant cover calculations are progressively decreasing, although the inflight VIS calibration target remains unchanged. To test the maximum effect of sensor degradation on plant cover values, we used the maximum sensor degradation factors for each band and manipulated the reflectance values prior to their transfer into plant cover values in the vegetation cover product. Afterwards, the differences in plant cover values were compared as calculated with and without manipulation of the reflectance values (Supplementary Fig. 2). Here, we found extremely small and thus negligible effects (root mean square error of below 0.8% plant cover).

### Precipitation and temperature data

Daily rainfall data that were provided by the Tropical Rainfall Measuring Mission (TRMM, 3B42) were used for the precipitation analysis^16^. The dataset was validated against field measurements and was successfully tested for its suitability to provide reliable estimates of rainfall variability^34^. The TRMM data have a spatial resolution of 0.25° × 0.25°, which was resampled to 20 km using the nearest neighbour approach. Resampling has been performed to use one single projection facilitating the comparison of the different datasets. The new spatial resolution was chosen to be close to the original one so that rescaling was kept to a minimum.

The 2-m air temperature values were derived from ERA-Interim reanalysis data^15^. This dataset has a spatial resolution of 0.75° × 0.75°, which was resampled to 60 km. The quality of the ERA-Interim air temperature values in the TP has been demonstrated by the very high correlations to data from meteorological stations^35^.

### Livestock numbers

LN for the provinces Qinghai and the TAR were taken from the official statistical surveys published in China Statistical Yearbooks^36^. Data for prefectures of the TAR were provided by the Tibet Statistics Bureau^37^. The absolute numbers of different animals were converted to sheep equivalents^38^.

### Time series analysis

The plant cover values were averaged over June, July and August (the growing season) because this period is characterized by vegetation growth across the entire TP^39^. Vegetation activity during the growing season is not necessarily influenced by only summer precipitation and temperatures. The winter precipitation may also be important for the greening-up of the C3 plant-dominated grasslands in the spring and thus may contribute substantially to plant growth in the early summer. Therefore, the inter-annual relationship between the grassland vegetation cover and climate factors was assessed for each pixel and climate dataset by a linear multi-temporal correlation analysis. Temporal lags from 0 to 6 months were tested at 1 month intervals (See Supplementary Fig. 3 for results of the linear multi-temporal correlation analysis). For each pixel, the climate data were aggregated for the lag period that corresponded to the time period that revealed the best correlation.

In each dataset, temporal trends across the entire time span of 14 years were calculated. For the vegetation cover, the values averaged over the summer period were used. For both climate datasets and each pixel, the average temperature values and precipitation sums of the time period were used which revealed the closest correlation to summer plant cover values. By dividing the differences between the time series and their mean values by their standard deviations, the anomalies of the plant cover and climate data were calculated for each pixel and dataset. The representativeness of the trends in the TRMM precipitation and ERA-interim temperature data for the Tibetan Plateau was assessed in a validation study against data from 49 meteorological stations (see corresponding section in the Supplementary material). The results of this validation study indicated that the climate trends are well captured by both data sets.

Trends within the anomalies were calculated separately for each pixel and dataset. Because most of the data were not normally distributed, the trends in the anomalies of the plant cover data and climate variables were calculated by the Mann-Kendall correlation techniques. We did not directly compare the time series of the different datasets in a multivariate analysis because of the coarser spatial resolution of the precipitation and temperature datasets compared to plant cover values.

## Additional Information

**How to cite this article**: Lehnert, L. W. *et al.* Climate variability rather than overstocking causes recent large scale cover changes of Tibetan pastures. *Sci. Rep.* **6**, 24367; doi: 10.1038/srep24367 (2016).

## Supplementary Material

Supplementary Information
