# Supplementary material for: Climate variability rather than overstocking causes recent large scale cover changes of Tibetan pastures
Source: Sci Rep. 2016 Apr 13;6:24367. doi: 10.1038/srep24367 (PMC4829870; doi:10.1038/srep24367)
Supplement: Supplementary Information [file srep24367-s1.pdf]

Supplementary material

Climate variability rather than overstocking causes recent large scale cover changes of Tibetan pastures

Lehnert, L. W., Wesche, K., Trachte, K. Reudenbach, C. and Bendix, J.

Supplementary Table S1: Pearson correlation statistics of the temporal trend analysis of temperature values (1980 - 2014). Data was taken from ERA-interim data and averaged for the area where thawing permafrost soils are considered as cause of increasing cover (33°N - 36°N, 86.25°E - 90.75°E). All variables were tested on normality.

Variable	Season	t	df	p value	r
Temperature	Year	3.33	34	< 0.01	0.50
	Winter	0.63	34	0.532	0.11
	Spring	2.20	34	0.035	0.35
	Summer	4.17	34	< 0.01	0.58
	Fall	1.82	34	0.078	0.30
No. of month with $\bar{t} > 0°C$		3.22	34	< 0.01	0.48

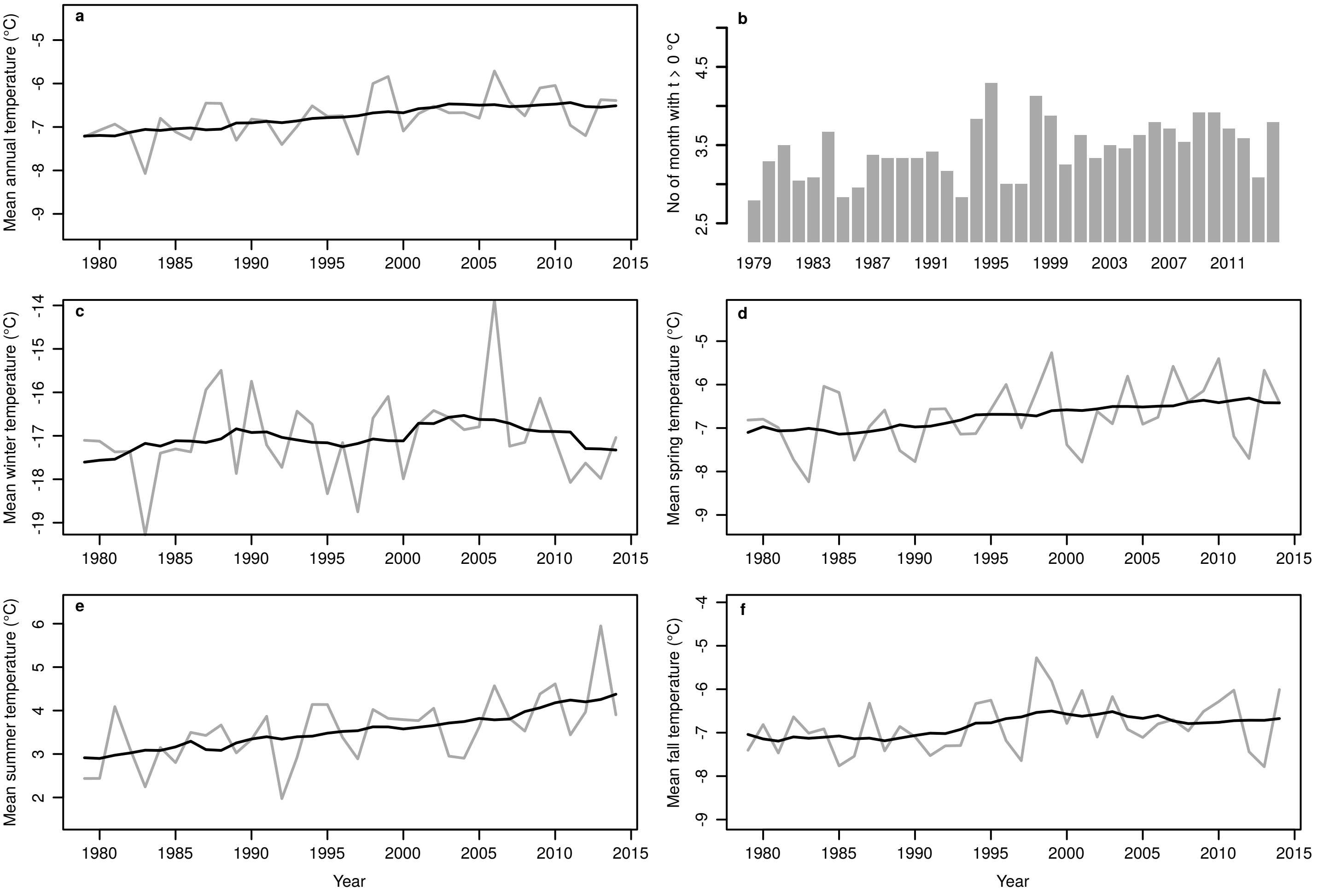

Supplementary Figure S1: Mean air temperature between 1980 and 2014 extracted from ERA-interim data for the area where thawing permafrost soils are considered as cause of increasing cover. (**a**) Mean annual temperature. (**b**) Number of months per year, where mean temperatures were above 0°C. (**c-f**) Mean temperature in winter (December - February), spring (March to May), summer (June - August), and fall (September to November). The grey lines are the mean values and the black ones the moving averages across 5 years. Spatial extent of the extracted data: 33°N - 36°N, 86.25°E - 90.75°E.

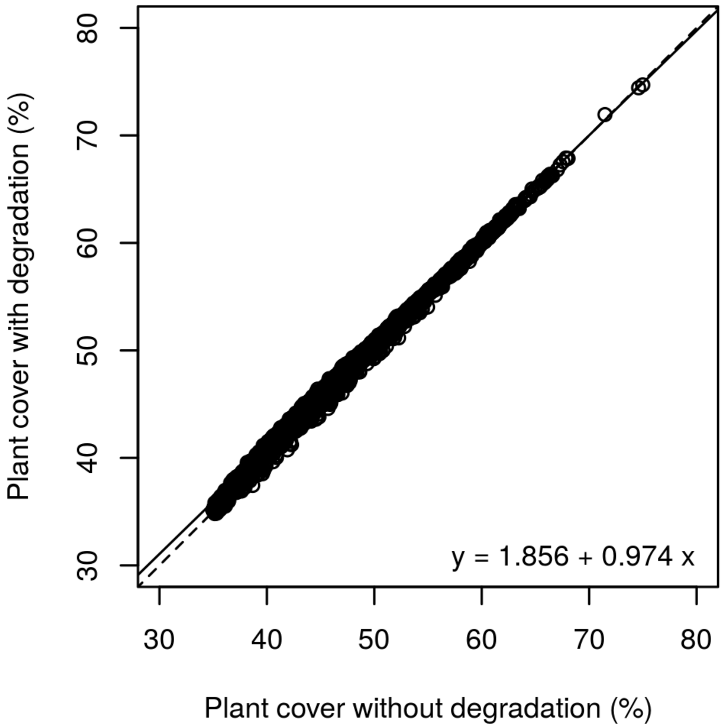

Supplementary Figure S2: Effect of sensor degradation on estimated plant cover values. The estimation is based on the maximum degradation factor of sensor degradation reported in 1. The dotted line is the 1:1 line, and the solid line is the linear regression between plant-cover values with and without sensor degradation.

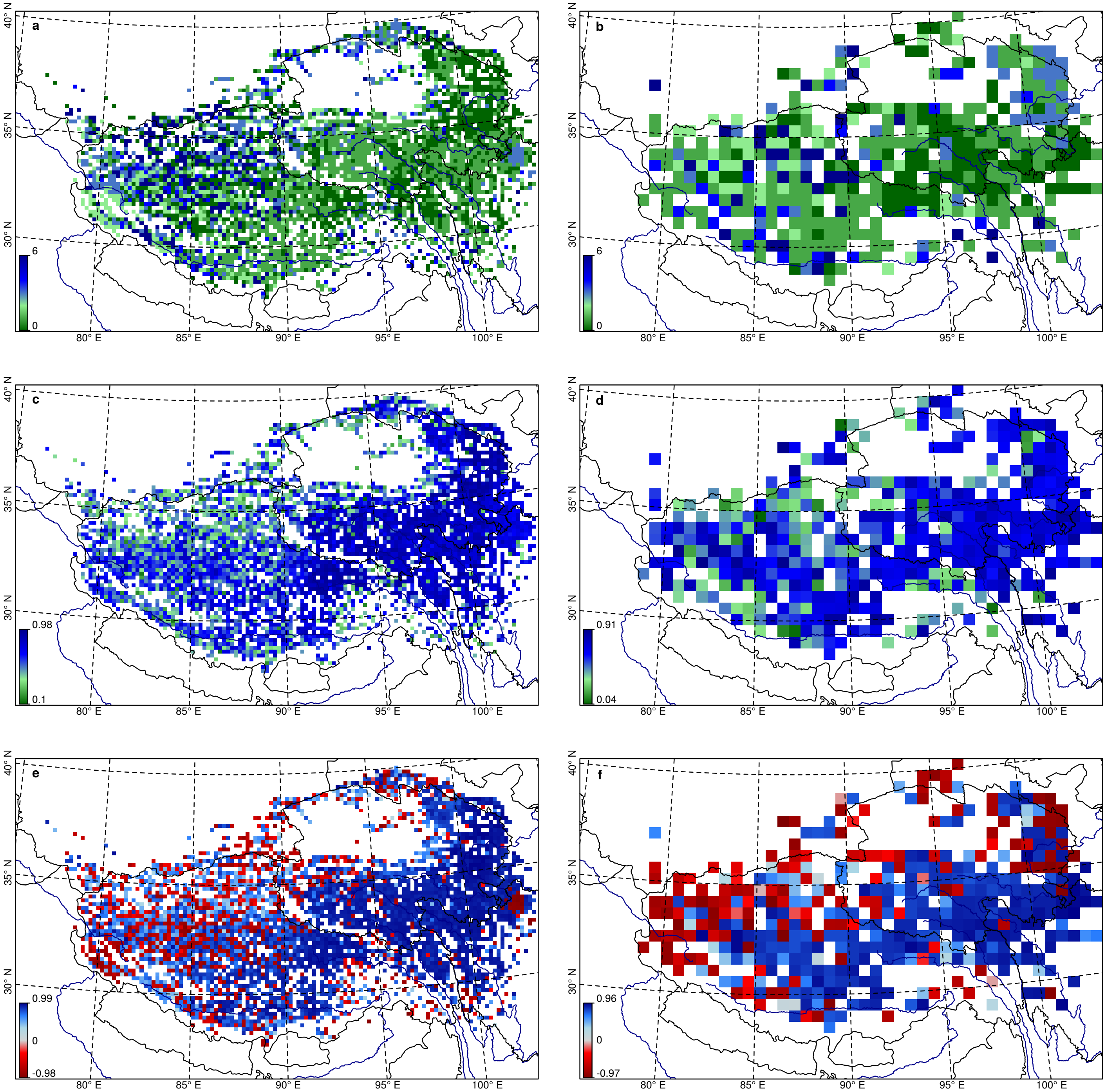

Supplementary Figure S3: Maps depicting relationships between plant cover and precipitation (left panel) and plant cover and air temperature (right panel) as revealed by the linear multi-temporal correlation analysis. (**a & b**) Time lags in months resulting in the maximum correlation coefficient; (**c & d**) absolute difference between maximum and minimum correlation coefficient observed for all tested time lags; (**e & f**) maximum absolute correlation coefficient. The maps have been created using R statistical software[2].

Validation of TRMM and ERA-interim trends

The accuracy of the precipitation and temperature data provided by the Tropical Rainfall Measuring Mission (TRMM, 3B42)[3] and ERA-interim reanalysis[4] was assessed to test if the trends in both data sets fit to those observed at meteorological stations. Therefore, the daily global historical climatology network data (GHCN-d)[5] were used which provided daily temperature and precipitation measurements from 49 meteorological stations spread over the entire Tibetan Plateau (Supplementary Fig. S4). The data was aggregated to the same time spans as the TRMM precipitation and ERA-interim temperature data sets (Supplementary Fig. S5). Afterwards, the precipitation sums and the mean temperature values of the TRMM and ERA-interim data have been compared to those measured at the meteorological stations by calculating the root mean squares error (RMSE) and the bias. Finally, to validate the trends in the precipitation data from TRMM and the temperature data from ERA-interim, Mann-Kendall correlation techniques were applied to the aggregated data sets.

The results indicated that the TRMM precipitation sums were in moderate agreement to the stations' data (Supplementary Tab. S2). High RMSE and BIAS values were observed at stations in the southern and south-eastern part of the Tibetan Plateau (Xigaze to Huili). Low errors were observed for stations in the Taklamakan basin where precipitation is generally extremely low (Kashi to Tikanlik). Spatial patterns for temperature errors were less pronounced. For instance, high errors were observed at Lhasa and Xigaze, again, but the closely situated station Xainza featured only a low RMSE value. The mean BIAS values for precipitation and temperature were 2.11 mm and -10.09°C, respectively. This indicated that precipitation is generally slightly overestimated by TRMM, whereas the mean air temperature simulated by ERA-interim is considerably lower than observed at ground. Irrespective of the variable, BIAS values did not follow any spatial pattern. However, it has to be kept in mind that certain uncertainties arise if spatial climate data is validated against data from meteorological stations. These uncertainties such as errors due to reduced representativeness may considerably enlarge the observed discrepancy between the compared data sets[6,7].

The comparison of the trends in the TRMM precipitation/ERA-interim temperature data against station data revealed that both data sets are able to capture the locally observed changes in climate elements (Supplementary Fig. S6). In general, both data sets tended to slightly underestimate the trends compared to those derived from meteorological stations. For TRMM precipitation and ERA-interim temperature trends, 7 and 5 stations indicated opposite trends, respectively (red points in Supplementary Fig. S6). Highest differences in Mann-Kendall τ-values between trends derived from TRMM and station data were observed in the Quaidam and Taklamakan basins which are not covered by grasslands of any of the considered types (e.g., Golmud, Dulan and Ruoqiang). In contrast, differences in areas with significant changes (e.g., southern Qinghai, stations Qumarleb to Guinan) were low. For temperature trends, high differences between trends from ERA-interim and station data were observed at the eastern declivity of the Tibetan Plateau. In this area, no significant temperature trend has been observed. In contrast, a good agreement between both data sets were observed for stations in the central part of the plateau (e.g., Nagqu, Xainza) where temperature changes are discussed as potential driver of vegetation changes.

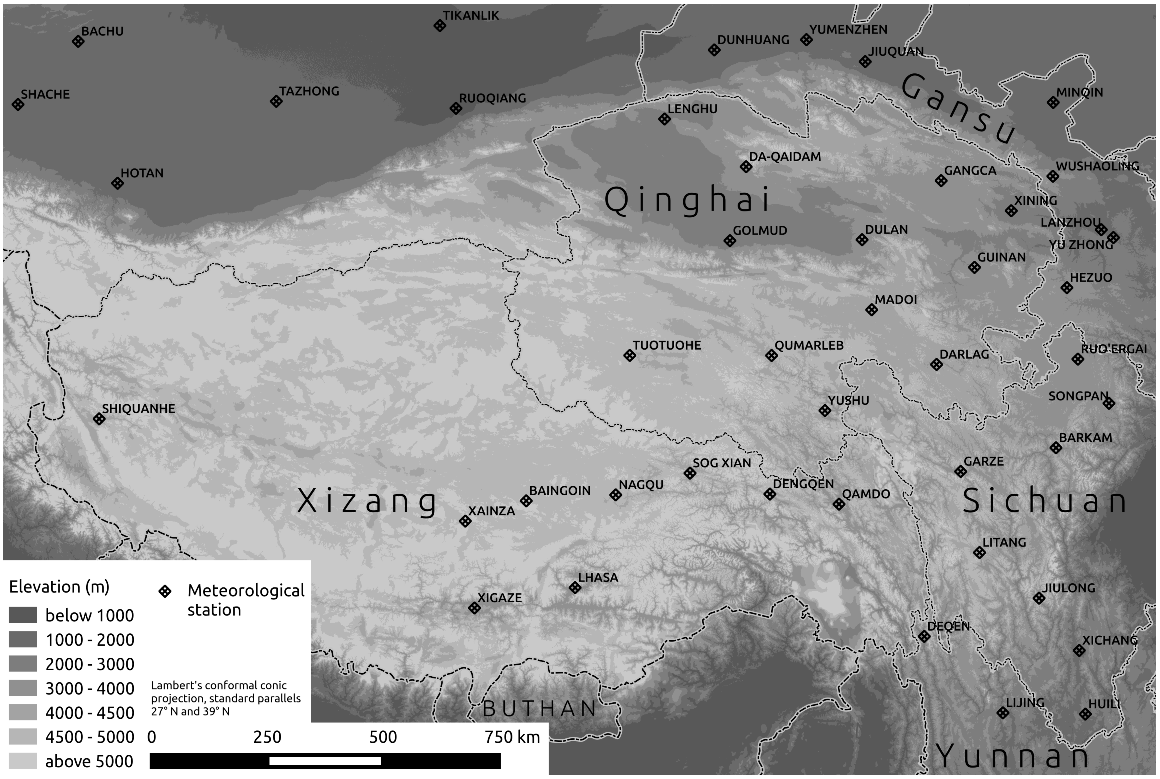

Supplementary Figure S4: Spatial configuration of meteorological stations used for validation of TRMM and ERA-interim data. Elevation data is from 8 and the map was created with QGIS[9].

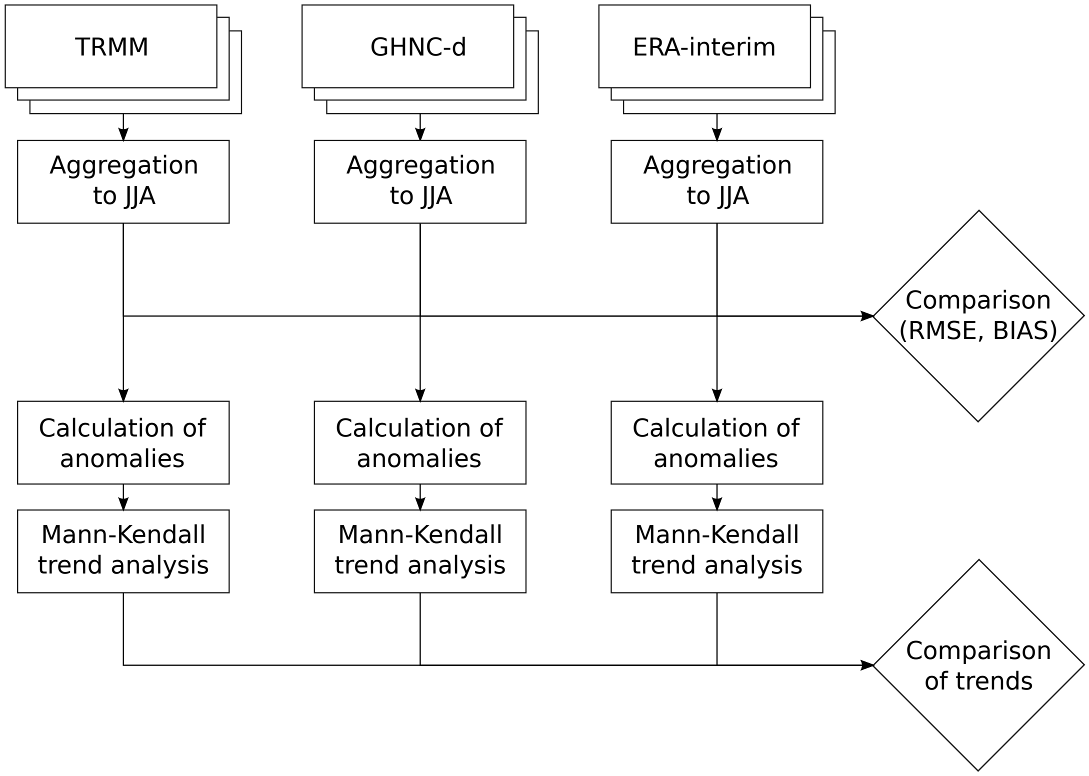

Supplementary Figure S5: Flow chart of validation process of TRMM and ERA-interim data against measurements of climate stations (GHCN-d).

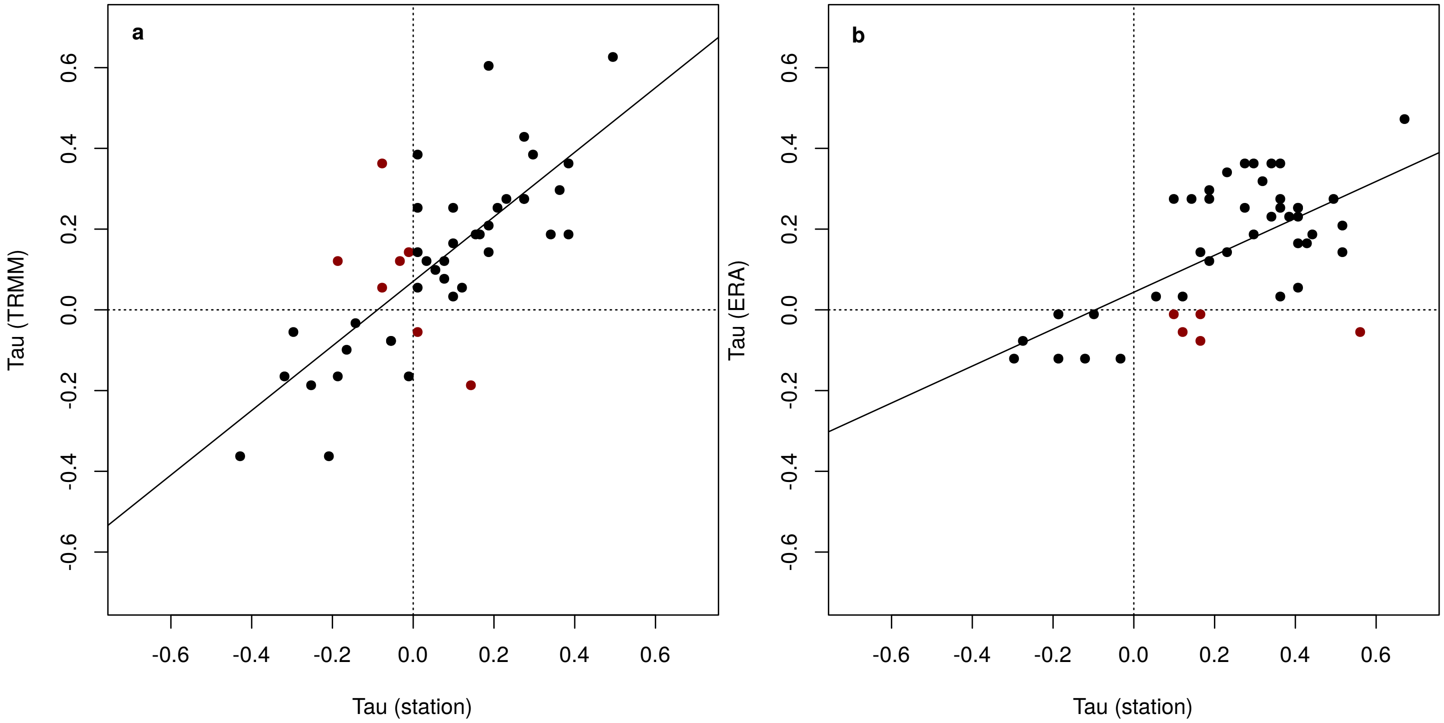

Supplementary Figure S6: Comparison of trends obtained from meteorological station data sets against TRMM precipitation (a) and ERA-interim temperature data (b). The dashed lines represent the zero trend lines and the solid ones are the linear regression lines. Red points represent those stations where the opposite trend between local data and spatial data was observed.

Supplementary Table S2: Summary of validation of TRMM precipitation sums and mean 2 m ERA-interim temperature data against meteorological stations. Stations are ordered alphabetically. TRMM and ERA-interim RMSE and BIAS values are in mm and °C, respectively.

Name	ID	Elevation	TRMM		ERA-interim	
		(m)	RMSE	BIAS	RMSE	BIAS
BACHU	CHM00051716	1117	4.79	0.56	7.17	-7.12
BAINGOIN	CHM00055279	4701	16.93	2.25	6.92	-6.81
BARKAM	CHM00056172	2666	16.12	7.53	16.57	-16.46
DA-QAIDAM	CHM00052713	3174	6.97	1.93	7.34	-7.21
DARLAG	CHM00056046	3968	12.26	-3.12	10.31	-10.18
DENGQEN	CHM00056116	3874	17.32	1.28	12.10	-12.03
DEQEN	CHM00056444	3320	27.47	8.88	4.99	-4.85
DULAN	CHM00052836	3192	14.09	-4.43	12.96	-12.92
DUNHUANG	CHM00052418	1140	4.76	-0.08	14.49	-14.28
GANGCA	CHM00052754	3302	18.49	-7.63	10.41	-10.33
GARZE	CHM00056146	3394	12.42	0.29	14.28	-14.18
GOLMUD	CHM00052818	2809	17.14	10.66	14.08	-14.03
GUINAN	CHM00052955	3123	13.76	-4.31	11.02	-10.98
HEZUO	CHM00056080	2910	13.05	1.65	9.99	-9.91
HOTAN	CHM00051828	1375	5.05	-0.94	12.68	-12.41
HUILI	CHM00056671	1788	24.05	-4.83	7.66	-7.59
JIULONG	CHM00056462	2994	20.31	7.10	11.65	-11.46
JIUQUAN	CHM00052533	1478	8.83	3.57	11.01	-10.92
KASHI	CHM00051709	1380	5.39	1.53	7.63	-7.51
KORLA	CHM00051656	933	6.37	-1.99	7.51	-7.40
KUQA	CHM00051644	1100	5.95	-1.20	6.48	-6.31
LANZHOU	CHM00052889	1518	14.11	2.72	9.95	-9.86
LENGHU	CHM00052602	2771	3.05	1.59	10.28	-10.24
LHASA	CHM00055591	3650	24.27	14.10	14.19	-14.11
LIJING	CHM00056651	2394	22.06	-2.33	8.44	-8.26
LITANG	CHM00056257	3950	15.42	-1.73	9.14	-9.04
MADOI	CHM00056033	4273	14.41	-1.87	8.50	-8.39
MINQIN	CHM00052681	1367	6.65	1.28	8.76	-8.68
NAGQU	CHM00055299	4508	19.93	6.69	8.70	-8.61
QAMDO	CHM00056137	3307	19.09	8.70	15.86	-15.78
QUMARLEB	CHM00056021	4176	13.04	2.56	10.03	-9.96
RUO’ERGAI	CHM00056079	3441	16.27	-1.10	6.90	-6.75
RUOQIANG	CHM00051777	889	5.30	1.02	11.04	-10.72
SHACHE	CHM00051811	1232	5.80	-0.77	6.26	-6.18
SHIQUANHE	CHM00055228	4280	13.32	8.36	14.38	-14.32
SOG XIAN	CHM00056106	4024	29.41	11.71	11.69	-11.61
SONGPAN	CHM00056182	2852	14.93	4.98	6.78	-6.53
TAZHONG	CHM00051747	1099	3.60	-0.22	8.20	-8.12
TIKANLIK	CHM00051765	847	3.23	0.37	7.78	-7.72
TUOTUOHE	CHM00056004	4535	11.21	1.77	8.63	-8.58
WUSHAOLING	CHM00052787	3044	13.99	-2.29	3.15	-2.81
XAINZA	CHM00055472	4670	20.98	8.32	9.00	-8.93
XICHANG	CHM00056571	1599	22.16	7.32	13.12	-12.94
XIGAZE	CHM00055578	3837	19.90	8.87	14.26	-14.17
XINING	CHM00052866	2296	11.47	-1.68	12.07	-12.02
YUMENZHEN	CHM00052436	1527	7.05	2.31	14.71	-14.56
YUSHU	CHM00056029	3717	12.89	3.81	12.40	-12.31
YU ZHONG	CHM00052983	1875	14.51	-2.04	8.23	-8.05

References in supplementary material

1. Wang, D. *et al.* Impact of sensor degradation on the MODIS NDVI time series. *Remote Sensing of Environment* **119**, 55–61 (2012).

2. R Core Team. *R: A Language and Environment for Statistical Computing (version 3.0.2)*. R Foundation for Statistical Computing, Vienna, Austria (2013). URL `http://www.R-project.org/`. (Date of access: 18/01/2016).

3. Huffman, G. J. *et al.* The TRMM multisatellite precipitation analysis (TMPA): Quasi-global, multiyear, combined-sensor precipitation estimates at fine scales. *Journal of Hydrometeorology* **8**, 38–55 (2007).

4. Dee, D. P. *et al.* The ERA-Interim reanalysis: Configuration and performance of the data assimilation system. *Quarterly Journal of the Royal Meteorological Society* **137**, 553–597 (2011).

5. Menne, M., Durre, I., Vose, R., Gleason, B. & Houston, T. An overview of the global historical climatology network-daily database. *Journal of Atmospheric and Oceanic Technology* **29**, 897–910 (2012).

6. Villarini, G., Mandapaka, P. V., Krajewski, W. F. & Moore, R. J. Rainfall and sampling uncertainties: A rain gauge perspective. *Journal of Geophysical Research: Atmospheres* **113**, 1–12 (2008). D11102.

7. Porcu, F., Milani, L. & Petracca, M. On the uncertainties in validating satellite instantaneous rainfall estimates with raingauge operational network. *Atmospheric Research* **144**, 73–81 (2014).

8. Natural Earth. Natural Earth. Free vector and raster map data. URL `http://www.naturalearthdata.com`. (Date of access: 18/01/2016).

9. QGIS Development Team. *Quantum GIS Geographic Information System. Open Source Geospatial Foundation Project (version 2.12)* (2015). URL `http://qgis.osgeo.org`. (Date of access: 18/01/2016).
